# Study of linear energy transfer effect on rib fracture in breast cancer patients receiving pencil‐beam‐scanning proton therapy

**DOI:** 10.1002/mp.17745

**Published:** 2025-03-18

**Authors:** Yunze Yang, Kimberly R. Gergelis, Jiajian Shen, Arslan Afzal, Trey C. Mullikin, Robert W. Gao, Khaled Aziz, Dean A. Shumway, Kimberly S. Corbin, Wei Liu, Robert W. Mutter

**Affiliations:** ^1^ Department of Radiation Oncology Mayo Clinic Phoenix Arizona USA; ^2^ Department of Radiation Oncology University of Miami Miami Florida USA; ^3^ Department of Radiation Oncology Mayo Clinic Rochester Minnesota USA; ^4^ Department of Radiation Oncology University of Rochester School of Medicine and Dentistry Rochester New York USA; ^5^ Department of Radiation Oncology Duke Cancer Institute Durham North Carolina USA; ^6^ Department of Pharmacology Mayo Clinic Rochester Minnesota SA

## Abstract

**Background:**

In breast cancer patients treated with pencil‐beam scanning proton therapy (PBS), the increased linear energy transfer (LET) near the end of the proton range can affect nearby ribs. This may associate with a higher risk of rib fractures.

**Purpose:**

To study the effect of LET on rib fracture in breast cancer patients treated with PBS using a novel tool of dose‐LET volume histogram (DLVH).

**Methods:**

From a prospective registry of patients treated with post‐mastectomy proton therapy to the chest wall and regional lymph nodes for breast cancer between 2015 and 2020, we retrospectively identified rib fracture cases detected after completing treatment. Contemporaneously treated control patients who did not develop rib fracture were matched to patients 2:1 considering prescription dose, boost location, reconstruction status, laterality, chest wall thickness, and treatment year. The DLVH index, *V*(d, l), defined as volume(*V*) of the structure with at least dose(d) and dose‐averaged LET (l) (LETd), was calculated. DLVH plots between the fracture and control group were compared. Conditional logistic regression (CLR) model was used to establish the relation of *V*(d, l) and the observed fracture at each combination of *d* and *l*. The *p*‐value derived from CLR model shows the statistical difference between fracture patients and the matched control group. Using the 2D *p*‐value map derived from CLR model, the DLVH features associated with the patient outcomes were extracted.

**Results:**

Seven rib fracture patients were identified, and fourteen matched patients were selected for the control group. The median time from the completion of proton therapy to rib fracture diagnosis was 12 months (range 5–14 months). Two patients had grade 2 symptomatic rib fracture while the remaining 5 were grade 1 incidentally detected on imaging. The derived *p*‐value map demonstrated larger *V*(0–36 Gy[RBE], 4.0–5.0 keV/µm) in patients experiencing fracture (*p *< 0.1). For example, the *p*‐value for *V*(30 Gy[RBE], 4.0 keV/um) was 0.069.

**Conclusion:**

In breast cancer patients receiving PBS, a larger volume of chest wall receiving moderate dose and high LETd may result in an increased risk of rib fracture.

## 1 INTRODUCTION

Proton therapy is an emerging modality for the treatment of breast cancer due to improved heart, lung, and other normal tissue sparing compared with photon techniques.^1^, ^2^ Modern pencil beam scanning proton therapy (PBS) provides even greater conformality of dose distributions than historical aperture and compensator‐based proton techniques, especially in the proximal portion of the beam path.^3^, ^4^, ^5^ This greater conformality of PBS enables improved organs at risk (OAR) sparing, particularly the skin, which is attractive for breast radiotherapy planning.^6^, ^7^ Therefore, PBS is increasingly utilized for the treatment of breast cancer.^1^

While cell killing with protons is primarily related to physical dose, it is also impacted by the proton linear energy transfer (LET).^8^ Protons, unlike photons, deposit most of their energy over a short distance at the end of the proton beam range, and there is evidence that the higher LET at the end of range may enhance the proton relative biological effectiveness (RBE).^8^, ^9^, ^10^, ^11^, ^12^ Enface or anterior oblique beams are typically used in breast cancer proton therapy planning. This beam arrangement enables planners to take advantage of the rapid energy deposition at the end of the proton range to maximize heart and lung sparing, and also reduces the sensitivity of dose conformality to respiratory motion.^13^, ^14^, ^15^, ^16^, ^17^, ^18^, ^19^, ^20^ With this beam distribution, the ribs and intercostal muscles, which lie immediately posterior to the breast, chest wall, and axillary clinical target volumes (CTVs) have the potential to be exposed to dose distributions with high dose‐averaged LET (LETd)^21^ and physical dose overlap. A rib fracture rate of up to 7% (grade 1 CTCAEv4.0) has been reported following proton therapy for breast cancer, higher than typically observed in the photon literature.^2^ The risk of rib fracture has previously been associated with LETd.^22^

Currently, a fixed RBE value of 1.1 is employed routinely in proton therapy which ignores the potential impact of spatial variation of LET. However, a RBE > 1.1 for adverse events (AEs) associated with higher LET within OARs has been reported for rib fracture,^22^ rectal bleeding,^23^ mandible osteoradionecrosis,^24^, ^25^ brain necrosis,^26^, ^27^, ^28^, ^29^ and late‐phase pulmonary changes.^30^ An improved understanding of the relationship between physical dose, LET, and AEs in proton therapy planning is greatly needed to improve treatment planning.

However, the clinical application of LET in PBS faces two major challenges. To calculate RBEs from LET and physical dose, several phenomenological RBE models^31^, ^32^, ^33^, ^34^ and mechanism‐related models^35^, ^36^, ^37^, ^38^, ^39^, ^40^ have been proposed. These models were parametrized and tuned by fitting in vitro measurements on clonogenic assays. Most of these models use tissue‐type related α/β ratio, which has large parameterization uncertainties.^32^, ^41^, ^42^ More importantly, enormous discrepancy of RBEs has been reported between in vitro and in vivo results.^43^ The results based on clonogenic assays are unlikely to represent clinical responses of cancer patients treated with PBS. Hence, RBE models are compromised by considerable biological and parametrical uncertainties, which have prevented the application of LET in clinical settings. Thus, it is important to directly use patient outcomes data and establish the empirical association between patient outcomes and the synergistic effects of dose and LET in PBS.

Dose‐LET volume histogram (DLVH) is a new tool that effectively combines the effects of LETd and dose in patient outcomes studies.^23^, ^44^, ^45^ Since DLVH is based on dose and LETd, instead of RBE, it strategically addresses the challenges of large uncertainties in the existing models of RBE.^8^ In this study, DLVH was employed to investigate the effects of dose and LETd on rib fracture risk in breast cancer patients treated with PBS‐based postmastectomy radiotherapy (PMRT). DLVH‐based statistical methodologies, including DLVH‐index‐wise fixed‐effect logistic regression modeling, were employed to reveal the dose/LETd patterns that are potentially associated with fracture risk.

## 2 METHODS

### 2.1 Patient cohort

This study was approved by our institution research board (IRB). From a prospective registry of post‐mastectomy patients treated with PBS to the chest wall and regional lymph nodes for breast cancer between 2015 and 2020, a radiation oncologist retrospectively identified rib fracture cases detected upon workup of chest wall pain or identified incidentally on imaging through chart review. Imaging was reviewed to confirm the identification and location of each rib fracture. Control patients without fracture were selected to match the fracture patients in a 2:1 ratio considering prescription dose, boost location, reconstruction status, laterality, chest wall thickness, and treatment year. This study was reviewed and approved by the IRB under protocol number 24‐011106.

### 2.2 Treatment planning and contouring

The CTV included the chest wall, levels I, II, and III of the axilla, supraclavicular nodes, and internal mammary nodes.^46^ The chest wall volume did not extend deeper than the anterior surface of the ribs and intercostal muscles (the ribs were not included in the CTV), and in reconstructed patients included the entire tissue expander or implant. The prescription dose to the chest wall and regional lymph nodes was either 50 Gy [RBE] in 25 fractions or 40 Gy [RBE] in 15 fractions. Simultaneous integrated boost (SIB) or sequential boost to lymph node and/or chest wall targets were used based on clinical risk factors at physician discretion. All patients were treated with multi‐field optimized (MFO) PBS plans using two to three fields, as previously described.^46^, ^47^, ^48^, ^49^ Treatment plans were generated in a commercial treatment planning system (Eclipse, version 15.1; Varian Medical Systems, Palo Alto, California, USA) using robust optimization^50^, ^51^ considering setup uncertainty of ± 5 mm and range uncertainty of ± 3%. For the CTV, the planning goals were D90% ≥ 90% (priority 1) under the worst‐case scenario of the plan robustness evaluation^52^, ^53^ D95% ≥ 95% (priority 2), and D0.01 cc ≤ 110% (priority 1).^49^ Treatment plans met the institutional dose volume constraints (DVCs) including target coverage and OAR dose constraints.^46^ In addition, all plans were assessed using an in‐house Monte Carlo biologic dose model.^54^ When clinically appropriate at physician discretion, attempts were made during plan optimization to limit areas of high LETd and high physical dose overlap on the ribs and intercostal muscles^47^ at the most posterior extent of the CTV. Plans were delivered using Hitachi PROBEAT‐V proton therapy system (Hitachi, Tokyo, Japan). Patients were treated under free‐breathing conditions. AlignRT (Vision RT, London, UK) was employed to monitor respiratory motion during treatment.^48^

### 2.3 Dose‐LET volume histogram (DLVH)

Recently, we have established the tool, DLVH, to study the associations of dose and LETd with normal tissue toxicity.^23^ To generate the DLVH, both dose and LETd for each voxel were considered. The dose and LETd were calculated using an in‐house Monte Carlo dose engine.^55^ This dose engine has been implemented as a second check, optimization, and biological dose evaluation platform for our clinical practice.^47^, ^54^, ^56^ LETd to medium for voxel at (x, y, z) was calculated based on the following:

(1) $$\text{LETd}\;\left(x,y,z\right)=\frac{\sum_{j}\phi_{E}\left(x,y,z,E_{j}\right)SP^{2}\left(E_{j}\right)\Delta E_{j}}{\sum_{j}\phi_{E}\left(x,y,z,E_{j}\right)SP\left(E_{j}\right)\Delta E_{j}},$$

where $\phi_{E}\left(x,y,z,E_{j}\right)$ is the energy spectrum of proton with an energy of $E_{j}$, and $SP(E_{j})$ is the unrestricted stopping power of protons with an energy of $E_{j}$ to the medium. For consistency, the dose reported in this study was also dose to medium.

DLVH index, *V*(d, l), was defined as the volume *V* (% for normalized volume or cc for absolute volume) of the chest wall structure with a dose of at least *d* Gy[RBE] and an LETd of at least *l* keV/µm, and was calculated [*V*(d, l) = *V*(Dose > *d*, LETd > *l*)]. We repeated this calculation process for all combinations of *d* and *l* within the dose and LETd ranges. A 3D volume surface plot was then established, which represents a joint cumulative histogram of dose and LETd distribution of the structure, in which dose and LETd are the two independent variables. For easy visualization, the 3D volume surface plot was then projected in the 2D dose and LETd plane as multiple iso‐volume lines, denoted as DL*v*%.

### 2.4 DLVH‐based analysis

**FIGURE 1:**
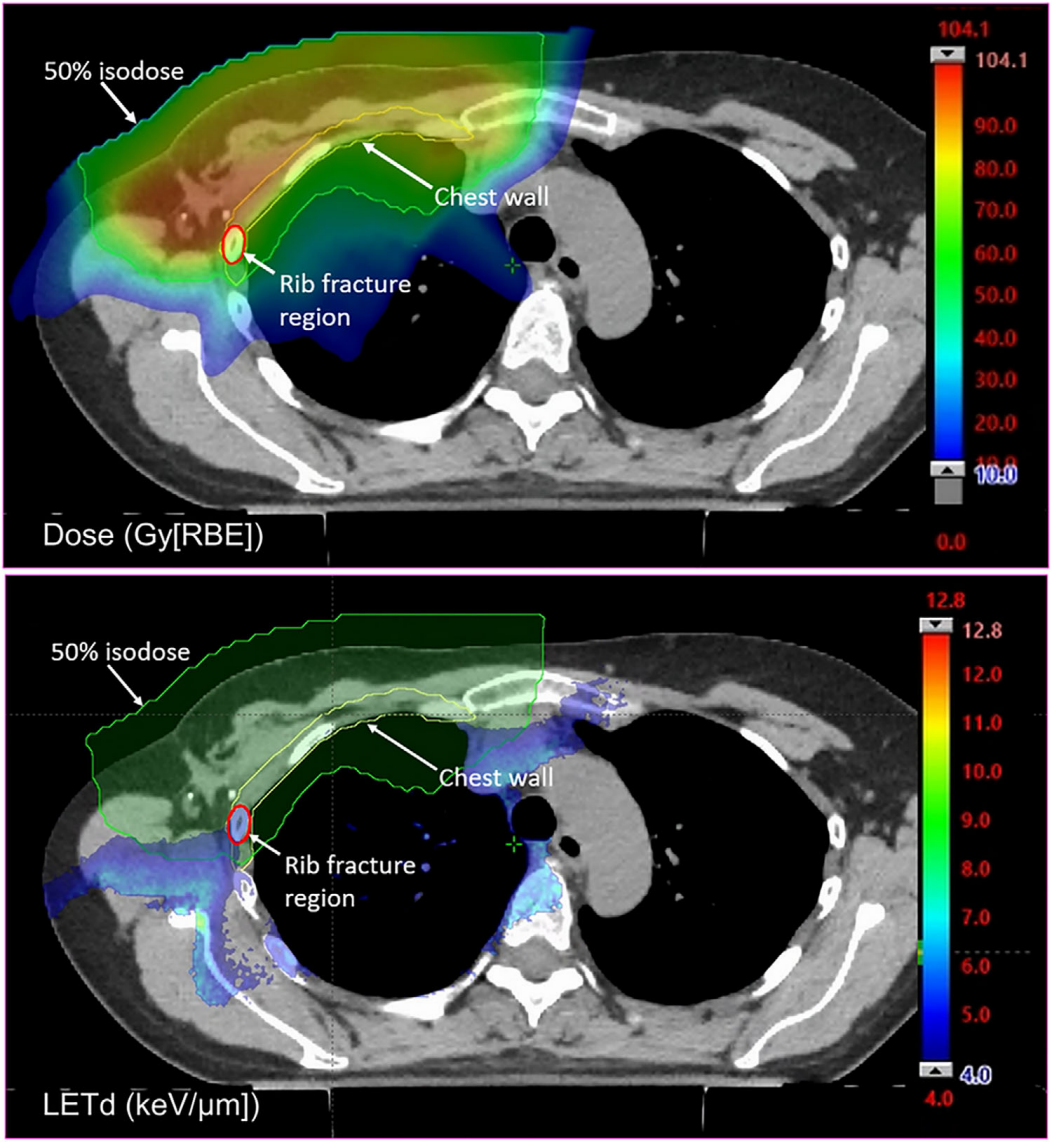
Dose (*top* panel) and LETd (*bottom* panel) distributions of one rib fracture patient. Contours represent chest wall (yellow), rib fracture region (red), and 50% prescription isodose line (green). The 100% prescription dose is 56.25 Gy[RBE]. LETd larger than 4 keV/µm is shown in color wash.

Chest wall DLVHs for all patients were calculated. The dose from hypofractionation plans were converted to conventional fractionation using EQD2 dose and α/β = 3. In this study, to evaluate the possible dose and LETd effect upon rib fractures, a risk‐associated chest wall structure was retrospectively contoured, which incorporated ribs and intercostal muscles enclosed by the 50% prescription isodose lines (Figure 1).

Conditional logistic regression (CLR) is extensively utilized to analyze case‐matched data, effectively mitigating the impact of the confounding factors. In this study, CLR was applied to the analysis. The volume index of DLVH, V(d, l), served as the independent variable and the binary outcome of rib fracture presented as the dependent variable.

Our CLR model incorporated two levels of effects influencing the outcomes: (1) Fixed effects. These accounted for systematic different clinical factors among rib fracture patients, such as prescription dose or the presence of tissue expander reconstruction. These factors were controlled by case‐matching, assuming these clinical factors are equivalent between the rib fracture patients and their matched controls. (2) Random effects. These were derived from the variations in the volume index of DLVH, V(d, l), between the rib fracture group and their matched control group. Therefore, mixed effects from both fixed and random factors were considered in this CLR model.

The model was expressed using the following equation:

(2) $$\text{logit}\;\left(\text{fracture}\right)=\alpha_{i}+\beta V\left(d,l\right)$$

where *i* was the indicator of the *i*th rib fracture patient, *β* was the regression coefficient, *α_i_* represented the fixed effect associated with the *i*th matched group. Logit was the inverse of the sigmoid function, used to model the probability of rib fracture.

The significance of the regression coefficient *β* is measured by its *p*‐value; a lower *p*‐value suggests a more substantial impact of the DLVH index on the likelihood of rib fracture.

For each combination of dose *d* (Gy[RBE]) and LETd *l* (keV/µm), a separate CLR model was established. To investigate all outcomes related DLVH indices and derive potential clinical insights, every possible DLVH index within the specified dose and LETd range was analyzed using CLR. This iterative process generated a *p*‐value map in the dose and LETd plane, where each *p*‐value, p(d, l), corresponds to the significance of *β* for each CLR model based on V(d, l). The *p*‐value map provides a transparent method for the identification and visualization of the typical DLVH features that closely associated with patient outcomes, thus enhancing our understanding of dosimetric risk factors in dose, LETd, and volume for rib fracture in breast cancer patients treated with proton therapy.

### 2.5 Statistics

DLVHs were calculated using Matlab 2019a (MathWorks, Inc., Natick, Massachusetts, USA). The fixed‐effect logistic regression was conducted using the generated “clogit” function of R (version 4.1.2). *p*‐values were obtained from the regression models and were plotted using Matlab. *p*‐value of < 0.05 was considered significant for this analysis.

## 3 RESULTS

We reviewed 216 patients with primary or recurrent breast cancer treated with proton PMRT, with a median follow‐up of 33 months (range 1–68 months) and identified 7 patients (i.e., 3.2%) who experienced rib fracture. The median time from the completion of proton therapy to rib fracture diagnosis was 12 months (range 5–14 months). Two had grade 2 symptomatic rib fracture, while the remaining five were grade 1 incidentally detected on imaging.

The clinical characteristics for the seven patients with rib fracture are displayed in Table 1. The median age at time of radiation treatment was 54 years (range 32–64 years). Six patients received a dose of 50 Gy[RBE] in 25 fractions to the chest wall and regional lymph nodes; of these, four received a SIB of 56.25 Gy[RBE] to the chest wall (*n* = 2) or axillary nodes (*n* = 2), and one received a sequential boost of 14 Gy[RBE] in 7 fractions to the chest wall. One patient was treated with 40 Gy[RBE] in 15 fractions to the chest wall and regional lymphatics on a randomized trial comparing conventional and hypofractionated PMRT (clinical trial number NCT02783690).^57^

**TABLE 1:** The patient and tumor characteristics for the patients identified with fractures.

Patient	Age	Stage	Fracture after RT (months)	Grade of toxicity	Boost^a^	Dose (Gy[RBE])	Fx	Topology	Laterality	Matched control #
1	39	IIIC	14	1	SIB	50/56.25	25	UIQ	L	2
2	63	IIA	11	2	No	50	25	LOQ	R	4
3	56	IIA	5	1	No	40.05^b^	15	UOQ	L	2
4	32	IIIC	14	2	SIB	50/56.25	25	UOQ	R	2
5	64	IIIC	14	1	SIB	50/56.25	25	LIQ	L	2
6	33	IIIC	11	1	Seq	50/64	25/32	UOQ	L	1
7	54	Recurrent	12	1	SIB	50/56.25	25	LOQ	R	1

Abbreviations: LIQ, lower inner quadrant; LOQ, lower outer quadrant; UIQ, upper inner quadrant; UOQ, upper outer quadrant.

^a^ SIB: simultaneous integrated boost.

^b^ 40.05: The doses from this hypofractionation plan were converted to conventional fractionation using EQD2 dose and α/β = 3.

As shown in Table 1, we could only identify one matched control for two patients because of unique clinical circumstances; one fracture case received a sequential boost which is infrequently performed in our practice, and the other case was treated for the relatively unusual presentation of recurrent disease. To maintain statistical power (i.e., the ability to detect a true effect if it existed) along with the overall 2:1 matching ratio between controls and cases, and to mitigate the potential impact of variability within the matched control patient subgroup, one of the cases was randomly selected from the other five patients to be matched with four control patients. The controls were selected based on prescription dose, boost location, reconstruction status, laterality, chest wall thickness, and treatment year. Thus, the entire cohort consisted of 7 fracture patients and 14 controls.

**FIGURE 2:**
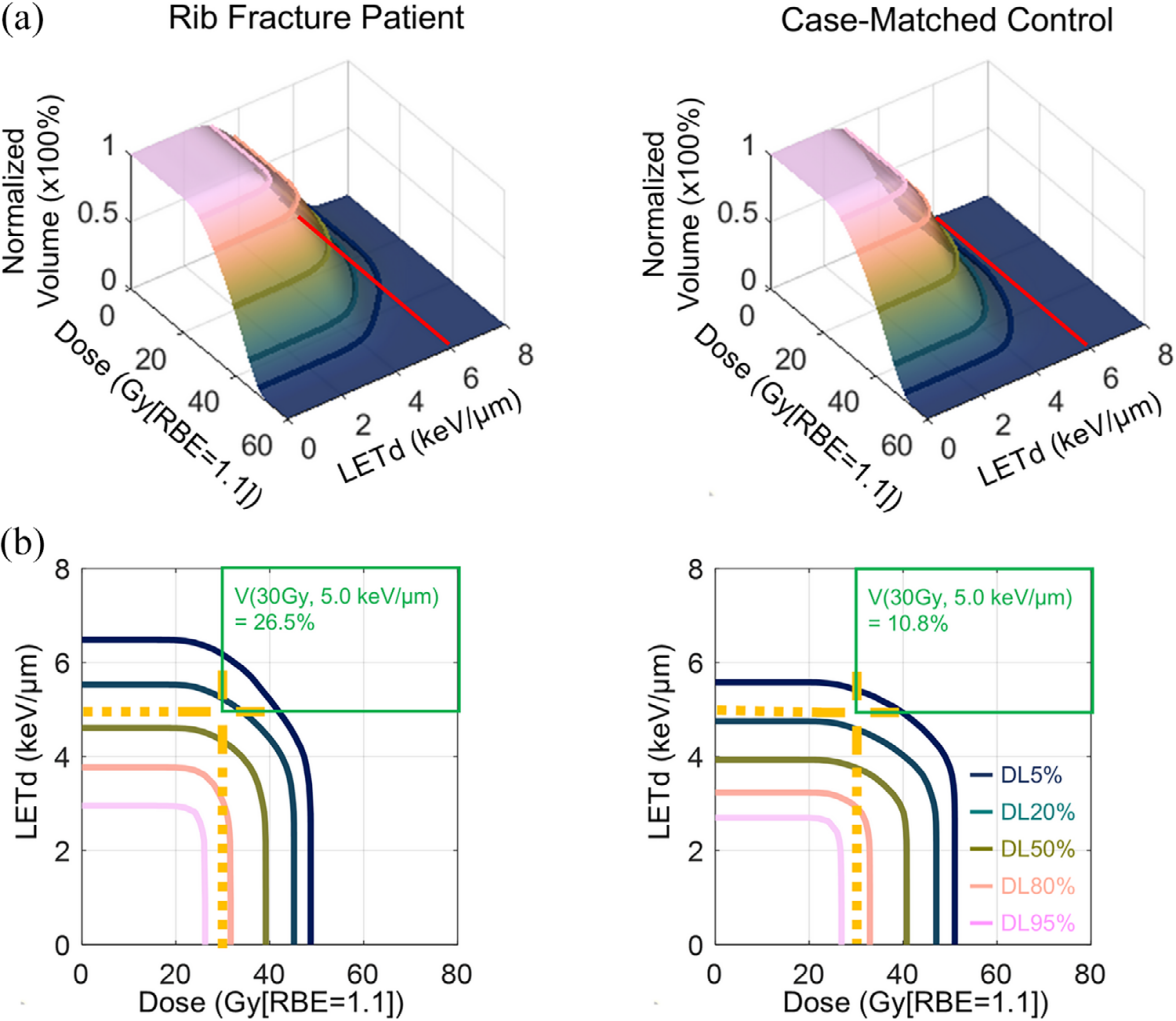
(a) 3D surface plot and (b) iso‐volume contour plot of Dose‐LET volume histogram (DLVH) of chest wall structure for one rib fracture patient (*left* panels) and one case‐matched control (*right* panels). Iso‐volume lines of DL5%, DL20%, DL50%, DL80%, and DL95% were displayed in the dose‐LETd plane. The DLVH index, *V*(d, l), was defined as *V*(% for normalized volume) of the structure with a dose of at least *d* Gy[RBE] and an LETd of at least *l* keV/µm. For example, the orange cross indicates the fractional chest wall volume of at least 30 Gy[RBE] and 5 keV/µm are 26.5% and 10.8% for fracture patient and the matched control, respectively. The red lines in Figure 2a are to highlight the difference of normalized volumes that received LETd > 6 keV/µm.

Figure 2a displays the 3D surface plots of DLVH from a representative fracture patient along with a matched control. In this plot, the normalized volume in the z‐axis represents the integral volume from both dose (x‐axis) and LETd (y‐axis). The volume at dose *d* Gy[RBE] and LETd *l* keV/µm represents the volume that has a dose of at least *d* Gy[RBE] and LETd of at least *l* keV/µm. The normalized volume thus forms a 3D surface, with unity volume at 0 Gy[RBE] and 0 keV/µm. Two red lines were drawn in Figure 2a to highlight the cross section between LETd of 6 keV/µm and the normalized volume. The rib fracture patient has larger volumes receiving high LETd (> 6 keV/µm) than the case‐matched control.

To better visualize the differences between fracture patient and the matched control, we contoured iso‐volume lines (5%, 20%, 50%, 80%, 95%) of DLVH plots and projected them to the dose‐LETd plane (Figure 2b). This helped us to observe the volume change relative to dose/LETd distributions. For example, in the facture patient (Figure 2b, *left* panel), 26.5% of the chest wall structure received at least 30 Gy[RBE] and 5.0 keV/µm, a point demarcated on the DLVH between the 20% and 50% iso‐volume lines by an orange cross. In contrast, only 10.8% of the chest wall received 30 Gy[RBE] and 5.0 keV/µm in the matched control (*right* panel), as demonstrated by the location of the cross between the 5% and 20% iso‐volume lines. Therefore, from the contoured DLVH plot, we were able to directly observe the differences in dose/LETd distributions by looking at the shift of the iso‐volume lines. DLVHs for all rib fracture patients and their corresponding matched controls are presented in the Supporting Information.

**FIGURE 3:**
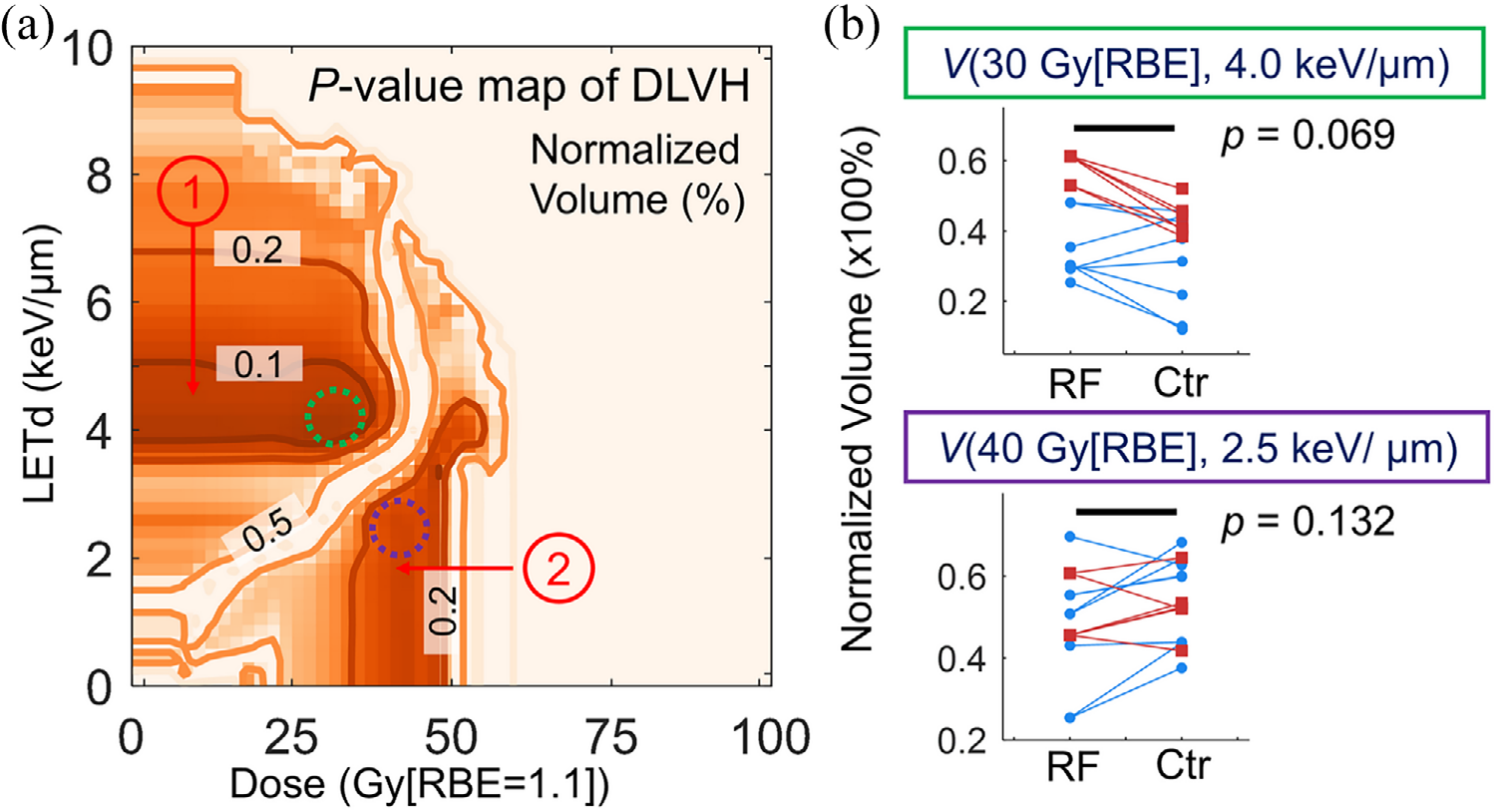
(a) *p*‐value map of CLR models for all DLVH indices. Iso‐*p*‐value lines of 0.1, 0.2, and 0.5 were contoured in the map. The features correlated with rib fracture are a larger *V*(0‐36 Gy[RBE], 4.0‐5.0 keV/µm) of the fracture patients (*p *< 0.1) (indicated as featured region 1 in red circled number). Another statistically less significant features (*p *< 0.2) are a smaller *V*(34‐48 Gy[RBE], 0–3.2 keV/µm) of the fracture patients (indicated as featured region 2 in red circled number). (b) Plots of two representative DLVH indices for the two features between fracture patients and the matched controls: DLVH index 1 (*top* panel): *V*(30 Gy[RBE], 4.0 keV/ µm), indicated as the dashed green circle in (a); DLVH index 2 (*bottom* panel): *V*(40 Gy[RBE], 2.5 keV/µm), indicated as the dashed purple circle in (a). The statistical *p*‐values are 0.069 and 0.132 for DLVH index 1 and index 2, respectively. For Figure 3b, the square and dot symbols represent patients with grade 2 and 1 rib fractures, respectively. Ctr, matched control patients; RF, rib fracture patients.

To determine which DLVH volume index is most strongly associated with rib fractures, we conducted mixed‐effect logistic regression analyses across all DLVH volume indices for a dose increment of every 2 Gy[RBE] and a LET increment of every 0.2 keV/µm. The resulting *p*‐values of the regression coefficients from all models were visually represented in a *p*‐value map in the dose and LETd plane. In this 2D map, one dimension corresponds to dose and the other to LETd. The corresponding *p*‐values were indicated by different color to create a two‐dimensional color map for better visualization (Figure 3a).

The *p*‐value map highlighted specific features associated with rib fractures, notably an enclosed area in the DLVH plot with a dose within 0–36 Gy[RBE] and a LETd within 4.0–5.0 keV/µm, denoted as V(0–36Gy[RBE], 4.0–5.0 keV/µm) (*p *< 0.1). This indicated that the volume receiving an intermediate physical dose and high LETd is greater in the rib fracture group compared to the control group.

For instance, the *p*‐value of the representative DLVH index, V(30 Gy[RBE], 4.0 keV/um) as shown in Figure 3b, was 0.069. Patients with grade 2 and grade 1 rib fracture were denoted by square and dot symbols, respectively, in the figure. It should be noted that due to the redundancy and interdependence among the DLVH indices, the *p*‐values with the nearby region with similar dose and/or LETd exhibited similar statistical significances.

Another feature, which was statistically less significant (*p *< 0.2), involved a smaller observed volume of dose within 34–48 Gy[RBE] and LETd of 0–3.2 keV/µm, for the rib fracture group (V(34–48Gy[RBE], 0–3.2 keV/µm), denoted as Feature 2 in Figure 3a. The *p*‐value for the representative DLVH index, V(40 Gy[RBE], 2.5 keV/um), presented in Figure 3b, was 0.132.

These findings suggested that the rib fracture group was subjected to a larger volume of intermediate physical dose and high LETd compared to the control group, yet they did not receive a high physical dose, which suggest the potential LETd‐enhancing effects upon rib fractures in breast cancer patients treated with proton therapy.

## 4 DISCUSSION

In this study, we utilize a novel DLVH‐based mixed‐effect logistic regression analysis method to identify LETd‐related dosimetric features that may be associated with rib fracture following proton PMRT. With these advanced methods, we found that larger volume of chest wall receiving high LETd and a modest physical dose was most associated with rib fracture. Our work adds to the growing body of literature suggesting that consideration of LETd is warranted during breast proton therapy planning and that LETd optimization may have the potential to further reduce the risk of rib fracture^2^, ^22^, ^47^ and other AEs of therapy.^30^

Rib fracture is a well‐known adverse effect of photon and proton‐based radiotherapy for breast cancer.^58^, ^59^ Some studies have suggested the possibility of increased rib fracture risk following proton therapy.^2^, ^60^, ^61^ Massachusetts General Hospital reported their initial experience of proton beam therapy for patients with breast cancer requiring regional nodal irradiation^2^; two‐thirds of patients were treated with PBS, whereas the remaining patients were treated with passively scattered proton therapy (PSPT). Of 70 included patients, five (7%) developed symptomatic or incidentally detected rib fracture. Verma et al. reported 2 of 91 (2%) patients experienced rib fracture after receiving proton beam therapy for regional nodal irradiation.^60^ The majority (77%) of patients were treated with PSPT, whereas the remainder received PBS. University of Florida recently reported their experience with 8 of 250 patients (3.7%) experiencing symptomatic or incidental rib fracture after proton radiotherapy for breast cancer.^61^ Of the included patients, 58% received PBS whereas the remainder were treated with PSPT or a combination of PBS and PSPT.

Recently, Gao et al. reported clinical outcomes for primary breast cancer patients treated exclusively with conventionally fractionated PBS.^46^ With a median follow‐up of 4.1 years, only two grade 2 (CTCAEv4.0) fractures from a total of 127 postmastectomy patients were reported. Recognizing limitations of cross study comparisons, the authors raised the possibility that the lower fracture rate could be due to differences in planning techniques. For example, all patients were treated with PBS using two or three fields, whereas for the patient cohort reported by Massachusetts General Hospital, the typical PBS treatment consisted of a single en face beam,^22^ which may lead to increased volume of high LETd on the chest wall at the end of proton beam range. In addition, efforts were made during Mayo Clinic Rochester treatment planning during latter years of this study to limit areas of overlapping high LETd and physical dose over the chest wall during treatment planning, facilitated by implementation of an institutional Monte Carlo biologic dose simulation that assumes a linear relationship between RBE and LETd.^47^ The current cohort of seven rib fracture cases includes the two rib fractures that were part of the manuscript by Gao et al.^46^ treated with conventionally fractionated proton PMRT. In addition, the current study includes one patient treated with hypofractionation on a randomized phase 2 trial comparing conventionally fractionated versus hypofractionated proton PMRT^57^ and four high‐risk patients with inflammatory breast cancer (*n* = 3) and recurrent breast cancer (*n* = 1).

In our study, we observed dosimetric features suggesting that fractures are primarily associated with higher LETd for the groups with matched physical doses. Our results are consistent with work by Wang and colleagues that a constant RBE model with a generic factor of 1.1 may be inadequate for predicting rib fracture risk.^22^ We observed LETd values of 4–5 keV/µm at moderate dose as a dominant feature. As illustrated in Figure 1, regions with LETd greater than 4–5 keV/µm are typically located outside the irradiated volume and thus receive relatively low doses. Conversely, regions with high doses are generally associated with lower LETd values. Therefore, rib fractures appear to be more closely associated with intermediate doses and LETd levels.

We observed a narrow valley region with the high *p*‐values. (approximately 25 Gy and 2 keV/µm to 45 Gy and 4 keV/µm). Based on the CLR model, the probability of rib fracture is predominantly influenced by clinical factors, making the contribution of dose‐LET volume indices in this valley region negligible. The shape of this valley region appears to be data‐dependent; however, it may represent a transition between different outcome‐related features.

Of note, the location of fractures may not be exactly in the regions of highest dosimetric risk. For example, Bradley et al. reported three fractures in patients treated with proton therapy for breast cancer that developed outside of radiation fields.^61^ Recent reports have suggested that regions of AEs evolve over time and can expand to include nearby voxels with low dose and low LETd.^25^, ^26^, ^28^ Therefore, in this study, we assessed the impact of LETd across the chest wall area receiving more than 50% of prescription dose, instead of focusing only on the site of fracture.^22^

A primary consideration for this choice also pertains to uncertainties in treatment delivery. Due to the small and variable structure of the ribs, accurately quantifying dose and LETd at these sites may be compromised by setup and range uncertainties in proton therapy. Additionally, the occurrence of rib fractures might not be solely a localized phenomenon. Complications could arise from hot spots outside the actual fracture sites or from general mechanical tensions. Consequently, we included both the ribs and intercostal muscles in our analysis to facilitate a more comprehensive volumetric assessment.

In the current optimization procedure, only voxels violating DVCs are penalized. LETd volume constraints could be implemented in a similar manner. However, dose and LETd volume constraints are applied separately, targeting different sets of voxels for penalization. This approach is suboptimal, as dose and LETd synergistically influence the initiation of AEs. To address this, we propose the introduction of the dose‐LET volume constraint (DLVC) within a robust optimization framework, hereby referred to as DLVC‐based robust optimization (DLVCRO).^62^ DLVC could be derived based on the DLVH methodology described in this manuscript. This advancement will transition proton treatment planning from a 2D to a 3D paradigm, considering dose, LETd, and volume to adjust both dose and LET distributions simultaneously. We anticipate that this refined method will effectively minimize AEs for patients. DLVCRO represents a promising strategy for future particle therapy treatments.

We utilized dose‐average LET in our DLVH analysis. While LETd is commonly used in clinical studies, its reliance on specific averaging models may reduce its effectiveness.^21^ Adopting heavy ion therapy field's approach of retaining particle spectrum information could enhance patient outcome modeling in proton therapy. It is important to standardize and harmonize the use and calculation of LET in the reporting of clinical outcomes and its implementation in proton therapy studies. Establishing consistent methodologies across studies is essential to ensure the comparability and reliability of results.

We used the DLVH volume index as the independent variable, instead of just dose and/or LETd. This approach offered several advantages: (1) it accounts for the volume effect, beyond just the numerical values of dose and LETd; (2) the interplay of dose and LET distributions within the organ is considered as we established the relation of clinical outcomes versus the DLVH index, *V*(*d, l*), the specific volume having both a certain dose and a certain LETd; (3) we phenomenologically derived the dose‐LETd relations based on patterns observed from the regression analysis as opposed to incorporating assumed dose‐LETd relations as variables into the regression analysis; and (4) DLVH maintains the integrity of LETd information, as the DLVH analysis allowed us to use patient cohort data at the organ level, while precisely investigating the LETd contribution. This avoided the data independency issue in the voxel‐based analysis.

Although uncertainties in RBE models require further investigations, they stem from three main sources: (1) data uncertainties from in vitro clonogenic assays used for model fitting; (2) parametric uncertainties, particularly with tissue‐specific α/β ratios; and (3) discrepancies between in vitro and in vivo results. While uncertainties in DLVH‐based modeling persist, they primarily arise from inter‐patient variability and clinical factors. The impacts of these confounding factors must be considered when studying patient outcomes. With a carefully designed study and the application of robust statistical methodologies, such as case‐matching and multi‐level conditional regression analysis as demonstrated in this manuscript, these uncertainties can be mitigated, allowing the actual effects of dose and LETd to be revealed.

DLVH analysis integrates dose and LETd information at the voxel level for histogram mapping. However, the actual dose and LETd distributions are highly sensitive to both setup uncertainties and respiratory motion. Given that the chest wall is typically located at the distal end of the treatment fields, even minor changes can result in significant variations in voxel‐level dose and LETd distributions, potentially compromising the accuracy of the DLVH analysis. Consequently, minimizing both inter‐ and intrafractional motions is critical. In this study, surface imaging was utilized to monitor chest wall motion, ensuring it remained within a 5 mm tolerance.^48^ For future studies, 3D robust evaluations that account for both nominal and uncertainty scenarios, or 4D evaluations that incorporate setup variations and respiratory motions using cumulative or dynamic dose/LETd distributions, will be crucial for improving the accuracy of DLVH analysis.

DLVH also has limitations: (1) Although voxel‐wise dose‐LETd relationship is preserved, the spatial information of the dose and LETd distributions is missing in the DLVH‐based regression analysis similar to the DVH‐based regression analysis for patient outcomes study; (2) because of small volumes at extreme dose and LETd, bin‐wise exhaustive search using DLVH indices at these regions are more prone to statistical errors. To address the first limitation, we will further investigate the geometric distributions of violating voxels. Moving forward, we will incorporate voxel mapping and local correlation into our analysis and further refine our model to obtain an optimized result at both the organ‐level and voxel‐level. The interpretation of modeling statistics at extreme doses and LETd needs to be approached with caution. However, given the redundancy of the DLVH indices, this error could be mitigated by examining other DLVH indices where their volumes are sufficiently accumulated.

There are several limitations to our study design. Post‐treatment screening for rib fracture is not routinely performed and thus the incidence of rib fracture may be underestimated. In addition, we use an institutional biologic dose model during treatment planning which could affect generalizability,^47^ and the parameters we identified may or may not be applicable to rib fracture risk following other indications for proton therapy such as reirradiation, whole breast, and partial breast irradiation where additional investigation is warranted.^1^, ^63^ Another limitation is that non‐dosimetric patient features that may be associated with rib fracture such as bone mineral density, receipt of chemotherapy, age, and menopausal status were not analyzed.^64^, ^65^ All these factors, along with dose and LET parameters, should be carefully evaluated in clinical practice. Further studies are needed to gain a better understanding of these clinical considerations and to address them effectively.

Of note, cases and controls from our study were drawn from one of the largest known proton PMRT institutional experiences in the world. Still, our analysis was limited by the small patient cohort due to the rarity of rib fracture in our patients. Although a high LETd volume effect was observed, the power was insufficient to demonstrate statistical significance or to perform a univariable analysis with other clinical characteristics. Notably, the volume metrics of DLVH in our analysis are highly dependent, reducing the conventional challenges associated with *p*‐value hacking seen with independent comparison pairs. Our transparent use of *p*‐value maps in feature selection aims to mitigate the risk of *p*‐value hacking. Where independent pairs occur, adjustments such as a tailored Bonferroni correction should be applied. Additionally, methods like Benjamini–Hochberg (BH) or Benjamini–Yekutieli (BY) procedures can help maintain statistical integrity. Pooling of data from multi‐institutional collaborators will be needed to further refine the predictive model. Toward these ends, we have initiated a multi‐institutional collaboration to aggregate more fracture cases after PBS. Through these collaborations, we hope to derive more conclusive insights to further optimize breast cancer proton treatment planning.

## 5 CONCLUSION

Our study reveals that larger volume receiving high LETd (i.e., >4 keV/um) and moderate dose may increase the risk of rib fractures in patients undergoing PBS PMRT. These preliminary results hold promise that DLVH can be translated into clinical practice. Integration of the derived DLVH features in treatment planning may potentially minimize the incidence rate of fracture and warrants further study.

## CONFLICT OF INTEREST STATEMENT

The authors declare no conflicts of interest.

## Supporting information



Supporting Information

## Data Availability

Research data are stored in an institutional repository and will be shared upon request to the corresponding author.
