# Supplementary material for: Study of linear energy transfer effect on rib fracture in breast cancer patients receiving pencil‐beam‐scanning proton therapy
Source: Med Phys. 2025 Mar 18;52(5):3428–38. doi: 10.1002/mp.17745 (PMC12059513; doi:10.1002/mp.17745)

Supplementary Materials

Figure S1

DLVHs (Dose-Linear Energy Transfer Volume Histograms) of 7 rib fracture patients and their matched controls.

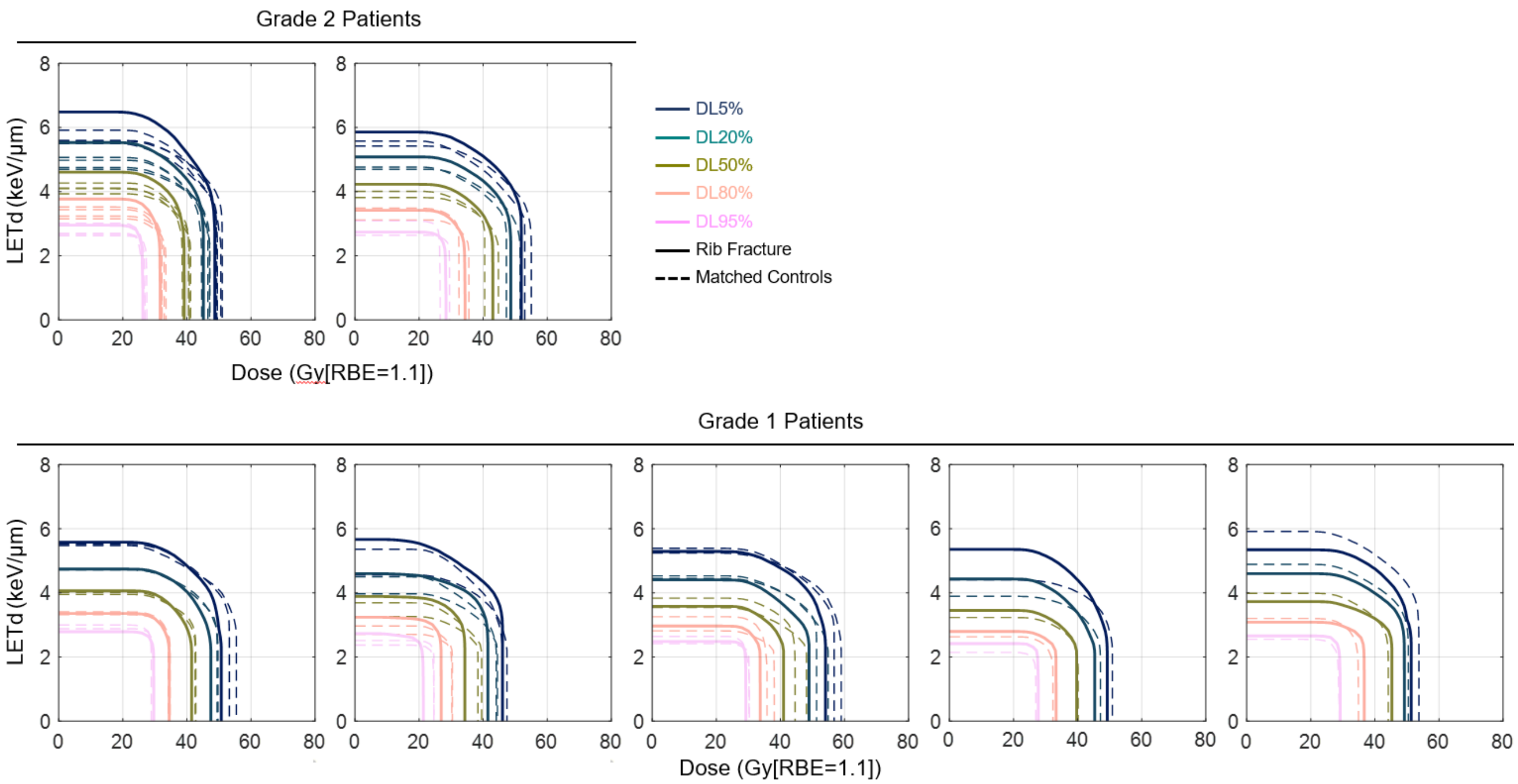

Supplement: Supplementary file 1 — Supporting Information [file MP-52-3428-s001.pdf]
